# Pathways and pitfalls: a qualitative study of student experiences in biomedical science education

**DOI:** 10.1002/2211-5463.70285

**Published:** 2026-06-08

**Authors:** Olivia J. Russell, Cassy Ross, Panagiota S. Filippou, Bruno Silvester Lopes, Dominic Gilchrist, Paul Dean, Kamar Ameen‐Ali, Tora K. Smulders‐Srinivasan, Ahmad A. Khundakar

**Affiliations:** ^1^ Population Health Sciences Institute, Faculty of Medical Sciences Newcastle University, Newcastle Biomedical Research Building, Campus for Ageing and Vitality UK; ^2^ School of Health & Life Sciences Teesside University Middlesbrough UK; ^3^ National Horizons Centre Teesside University Darlington UK; ^4^ Laboratory of Biological Chemistry, Faculty of Health Sciences, School of Medicine Aristotle University of Thessaloniki Greece; ^5^ Faculty of Medical Sciences, Translational and Clinical Research Institute Newcastle University UK

**Keywords:** biomedical science, Education, qualitative research, student experience, widening participation

## Abstract

Innovative pedagogies and support systems are critical for engaging diverse students in biochemistry and molecular biology education. This qualitative study explored biomedical science students' experiences of teaching, learning and support at a UK post‐92 university through surveys and focus groups (*n* = 15). Reflexive thematic analysis revealed three themes. Students' motivations were shaped by scientific curiosity and career aspirations in bioscience. Barriers to progression included financial strain, pandemic‐related disruption to laboratory access and cultural exclusion. Support and belonging were mediated through peer networks, while institutional support was inconsistently accessed and curriculum gaps limited experiential skill development in practical laboratory work. The findings underscore the need for pedagogies that blend active, experiential learning with codesigned curricula and integrated support. Such innovations are crucial for creating inclusive bioscience learning environments, improving retention and addressing structural inequalities in science education. These insights provide actionable guidance for biochemistry and molecular biology educators seeking to enhance student engagement and success.

## Graphical abstract

Biomedical science students from underrepresented backgrounds face barriers including financial strain, disrupted laboratory access and cultural exclusion. Peer networks provide vital support when institutional systems are difficult to navigate. To create inclusive learning environments and achieve academic success, educators should blend active, hands‐on pedagogy with codesigned curricula and responsive support systems.

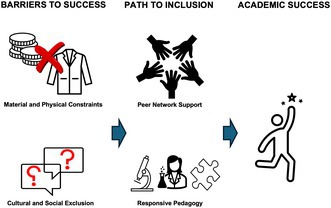

**Abbreviations**
COREQ: Consolidated Criteria for Reporting Qualitative Research
COVID‐19: coronavirus disease 2019
HCPC: Health and Care Professions Council
IBMS: Institute of Biomedical Science
MS Teams: Microsoft teams
NHS: National Health Service
NVivo: qualitative data analysis software
STEM: science technology engineering and mathematics
UK: United Kingdom

Widening participation in higher education remains a national priority [1], yet students from disadvantaged and underrepresented backgrounds continue to face persistent inequalities in access, retention and progression [2, 3]. In biomedical science, these disparities undermine efforts to build a diverse and representative healthcare workforce [4]. Structural disadvantage, racism, social class and financial pressures shape STEM students' trajectories, producing uneven learning experiences and limiting opportunities for advancement [5, 6, 7]. For disciplines such as biochemistry and molecular biology, which require mastery of complex scientific concepts, development of laboratory skills and the ability to connect theoretical knowledge to practical application, these barriers can be particularly consequential, yet the ways in which students from widening participation backgrounds navigate these disciplinary‐specific challenges remain underexplored.

A diverse biomedical science workforce enhances cultural competence, reduces health disparities and supports scientific innovation ([8]; NHS [9, 10]). Post‐92 universities (former polytechnics granted university status after 1992) in the United Kingdom have been central to widening participation, enabling higher proportions of first‐generation, mature and working‐class students to access higher education [11]. However, sector‐wide challenges persist, including unequal access to resources, limited academic and professional networks, and variable institutional support [12, 13]. A lack of visible role models and culturally inclusive curricula can also contribute to feelings of exclusion among minoritised students [14, 15]. These issues are often intensified in postindustrial regions with low social mobility and persistent deprivation [16, 17].

Within biomedical science education specifically, students from widening participation backgrounds encounter additional disciplinary challenges. These include navigating the specialised language of the biosciences, developing competence in laboratory techniques, and accessing experiential learning opportunities, such as placements and research internships, which are increasingly critical for graduate employability [18, 19]. Laboratory‐based disciplines demand practical skills difficult to replicate outside authentic environments [20]. When access to laboratories was disrupted during the COVID‐19 pandemic, the resulting gaps in experiential learning had enduring consequences for students' confidence and career readiness [21], effects that may be amplified for those already navigating structural disadvantage.

Despite growing recognition of these challenges, the lived experiences of biomedical science students in universities with a strong widening participation mission, including many post‐92 institutions beyond the Russell Group (the UK's 24 research‐intensive universities) in the United Kingdom, remain comparatively underexplored [22, 23]. This qualitative study therefore examines the social, financial and structural barriers faced by biomedical science students at different stages of their studies in a post‐92 university located in a post‐industrial region of the United Kingdom. While the sample includes students from diverse backgrounds, the institutional context means findings are particularly relevant to understanding the experiences of students from widening participation backgrounds.

By centring student voices, it reveals how systemic factors shape educational trajectories and constrain progression. While focussed on biomedical science, the discipline serves as a lens for examining wider challenges in bioscience, including mastery of scientific discourse, access to laboratory learning and development of professional identity. This study contributes to the scholarship of bioscience education by examining how students experience teaching practices, curriculum design and support systems, offering evidence to inform more engaging, inclusive and pedagogically sound approaches.

## Methods

This study employed a qualitative research design, using reflexive thematic analysis [24, 25], which was chosen for its suitability in providing a rich, nuanced exploration of participants' lived experiences and for its systematic yet flexible approach to identifying, analysing and interpreting patterns within the data. The research was situated within a pragmatic philosophical framework. Ontologically, university life was understood as multiple and socially constructed, with no single perspective offering a complete picture. Epistemologically, knowledge was approached as cocreated through interaction between researchers and participants. Focus groups were selected as the primary method to capture this diversity, allowing participants to reflect collectively and reveal both shared and contrasting experiences of social, financial and structural barriers in higher education. The study was conducted and reported in accordance with the Consolidated Criteria for Reporting Qualitative Research (COREQ) 32‐item checklist [26] to ensure methodological rigour and transparency (see File S1).

### Researcher reflexivity and positionality

The research team comprised five female and four male academic staff (lecturers and course leaders) and a postgraduate student from the host university. All facilitators held PhDs in bioscience or education‐related fields, except for the first author, who was a Master's in Public Health student at the time of the study. The team's primary expertise was in the biomedical sciences, and while experienced in quantitative research, their formal training in qualitative methods was developing, a limitation acknowledged and actively managed throughout the research process. The facilitators had pre‐existing academic relationships with participants as their lecturers, course leaders or personal tutors. To manage the potential influence of this power dynamic and the research team's assumptions as educators, several strategies were employed: the use of a neutral, open‐ended topic guide; regular debriefing sessions to challenge interpretive assumptions; and maintenance of a reflexive audit trail. Participants were informed of the researchers' roles and the study aims via the Participant Information Sheet.

### Participant selection and recruitment

Participants were recruited from the BSc (Hons) Biomedical Science and MSc Advanced Biomedical Science programmes at Teesside University, both accredited by the Institute of Biomedical Science (IBMS). This accreditation means graduates can train as Health and Care Professions Council (HCPC)‐registered Biomedical Scientists in the National Health Service (NHS) and elsewhere, which is relevant to interpreting their motivations and concerns about employability. A purposive sampling strategy was used to ensure representation across different academic stages, with one focus group conducted per undergraduate year and one with MSc students. Potential participants were approached via email invitations sent by their course leaders, who acted as gatekeepers. The invitations included a Participant Information Sheet, an electronic consent form and a link to a presession demographic survey. Fifteen participants provided informed electronic consent, which covered audio recording, transcription, confidentiality, secure data storage and publication of anonymised findings. A further seven students who expressed initial interest did not attend the scheduled sessions; no specific reasons were recorded, though scheduling conflicts are presumed.

### Data collection

Data collection occurred from March to April 2025. Four online focus groups were conducted via video conference on MS Teams, a format chosen to facilitate participation across cohorts and accommodate students' varied schedules and caring responsibilities. Only the participating students and the facilitating researchers were present. Each session lasted approximately 45 min, was audio‐recorded with consent, and was facilitated by a member of the research team using a semi‐structured topic guide (File S2). The guide covered key domains: motivations for studying biomedical science; experiences of institutional support; financial pressures; engagement with teaching and learning, including laboratory‐based practicals and career preparedness, which included motivation‐related prompts (File S2). One researcher led the discussion while another took observational notes on group dynamics and non‐verbal cues. Prior to each session, participants completed a brief online survey collecting anonymised demographic and background data.

### Data analysis

Audio recordings were transcribed verbatim by a professional transcription service, checked for accuracy by the research team and anonymised using a structured identifier system (e.g. Y02‐01 for Year 2, Participant 1). Data analysis followed Braun and Clarke's six‐phase framework for reflexive thematic analysis [24, 25], assisted by the nvivo 15 software. The process was inductive, with themes derived from the data rather than imposed through pre‐existing theories. Two researchers independently coded a subset of transcripts to establish analytical consistency, after which the lead researcher coded the full dataset. Codes were iteratively reviewed, clustered and refined by the research team to develop the final thematic structure. Thematic saturation was considered achieved as discussions across the four groups yielded consistent and recurring patterns, with no new themes emerging in the final group. Transcripts were not returned to participants for comment.

### Ethical considerations

Ethics approval was granted by Teesside University School of Health and Life Sciences (Life Sciences) Ethics Committee on 13 March 2025 (REF: 27229). All participants provided informed electronic consent, which covered audio recording, transcription, confidentiality and secure data storage. Procedures were in place for the secure handling of all data and for safeguarding in case of disclosures of distress or risk. All data were stored on a password‐protected, university‐approved server in accordance with institutional data protection policies. The study was conducted in accordance with the ethical standards outlined in the 1964 Helsinki Declaration and its later amendments.

## Results

### Presession questionnaire

The prefocus group questionnaire revealed a demographically diverse sample with varied educational and socio‐economic backgrounds (Table 1). The sample included students from a range of socio‐economic backgrounds, with 33% identifying as first‐in‐family to attend university and 20% as international students. Participants commonly reported financial pressures, part‐time work and reliance on financial support. Key barriers included limited mentorship, networking opportunities and affordable housing, with socio‐economic background influencing access to opportunities in biomedical science. Tutor and lecturer support was identified as the most valued form of assistance.

**Table 1:** Participant demographics.

Category		Count (*n* = 15)	Percentage
Age	18–24	8	53%
	25–34	6	40%
	35–44	0	0%
	45 and above	1	7%
Gender	Male	5	33%
	Female	10	67%
Ethnicity	White	8	53%
	Black or Black British	1	7%
	Asian or Asian British	4	27%
	Mixed Ethnic Group	0	0%
	Other	1	7%
	Prefer not to say	1	7%
Year of study	Year 1 (Undergraduate)	2	13%
	Year 2 (Undergraduate)	2	13%
	Year 3 (Undergraduate)	7	47%
	Master's	4	27%
Origin	Local	10	67%
	National (rest of UK)	2	13%
	International	3	20%
First in Family at University	Yes	5	33%
	No	10	67%
Part‐time employment during term	Yes, part‐time (< 20 h/week)	9	60%
	Yes, full‐time (≥ 20 h/week)	1	7%
	No	5	33%
Financial difficulties affecting studies	Yes, significantly	5	36%
	Yes, somewhat	4	29%
	No	5	36%

Open‐ended responses indicated strong motivations rooted in a passion for science, health care and making a societal impact, including interests in clinical investigation, medical research and laboratory work. Participants highlighted aspirations for well‐paid, impactful careers and hands‐on scientific application. Commonly cited nonacademic barriers included limited employability support, access to lab experience, internships and clinical placements, as well as challenges balancing academic, work and personal responsibilities, financial stress and local job scarcity. Suggested support measures included structured mentorship, enhanced networking, flexible payment plans and increased focus on practical, clinically relevant training.

### Focus group analysis

Thematic analysis of the four focus groups revealed three interrelated themes: Motivations and Pathways, Barriers and Pressures, and Support and Belonging. These highlight biomedical science students' complex educational journeys, shaped by personal aspirations, structural and financial constraints and the presence or absence of support systems.

### Theme 1: motivations and pathways

Students described a range of motivations for pursuing biomedical science, often combining intrinsic curiosity with pragmatic considerations. For many, an affinity for the subject provided a foundation for sustained engagement. One participant, who expressed a deep love for microbiology, also reflected on how this passion evolved into a clearer career direction:

I really love microbiology and pathology and I love to see how the world works, basically. (Y01‐02)

This intrinsic curiosity, she explained, gradually steered her from initial medical aspirations towards a research‐focused pathway:

Initially I was always into studying medicine, but then I realised I preferred the research side. (Y01‐02)

Others were drawn by the intellectual challenge and desire for continuous learning, an orientation that requires nurturing through appropriate pedagogical approaches:

It is fascinating and you learn things all the time… I love to challenge myself. (Y01‐01)

Pragmatic considerations also featured prominently, with some students valuing flexibility as a strategic response to an uncertain graduate labour market, as one student remarked:

I'm not very good at choosing my path per se, so biomedical science opens a lot of doors. (Y02‐01)

For several participants, entry into higher education itself was transformative. One mature student described her unplanned but deeply meaningful path to university:

Mine was a bit of a happy accident, but I'm really glad it happened. (Y03‐03)

Another student explicitly credited a foundation year with validating her sense of belonging and transforming her educational trajectory:

I did a foundation year and then I chose BioMed. If I'm honest, it was the biggest blessing. (Y03‐04)

Early exposure to healthcare settings shaped some participants' career choices, providing crucial insight for students without family connections in science:

I used to volunteer at our hospital… and I think BioMed is one of the roles that I've chosen. (Y01‐01)

The same student expressed a desire to apply scientific knowledge for broader societal benefit:

My motivation comes from wanting to make a positive impact in the world using science to help people. (Y01‐01)

### Theme 2: barriers and pressures

Despite evidence of strong motivations among students, many described multiple, intersecting barriers that affected their progression. The lasting impact of COVID‐19 was evident in accounts of inadequate transition support, with one student expressing a sense of institutional abandonment:

It was like the pandemic had never happened… My third‐year grades took a massive hit. (Y04‐03)

Financial pressures were pervasive, particularly for students with additional constraints. For international students, visa restrictions intersected with financial need, creating barriers not faced by domestic students:

We can only do like 20 hours per week… that's a big challenge. (Y01‐01)

For students with caring responsibilities, pressures intensified dramatically. One parent described the difficulties of childcare, illness, financial precarity and academic deadlines:

With four children, when they're passing illnesses around it's hard. They want me at home but I still have to get to uni, and financially we live week to week. (Y03‐02)

Experiences of marginalisation were recounted by several participants. One student described a class‐based microaggression at a previous institution giving a sense of inferiority:

They're like, ‘I didn't know we let people in from where you're from??’…. (Y04‐03)

The same student described gender‐based discrimination at the same previous institution reflecting stereotypes about women's capabilities in STEM:

I've been told I've done ‘good for a girl’ by a professor. (Y04‐03)

For international students, technical jargon posed challenges distinct from everyday English proficiency. One student highlighted this ‘hidden curriculum’ element of academic discourse:

It's not the English that's the problem, it's the scientific language. (Y01‐02)

Housing instability affected one international student significantly, revealing how this derailed academic progress:

The whole moving about sounds fun at first… But in reality, it's not very nice. My studies were disrupted completely. (Y02‐02)

Another student elaborated on the explicit barriers faced by those who had not previously worked in a laboratory:

If you haven't physically been working in an industry lab, they won't even consider your application. (Y03‐02)

### Theme 3: support and belonging

Students described a stark contrast between available institutional support and their ability to access it. The hidden curriculum that advantages students with greater cultural capital was evident in one student's confusion about navigating support services:

I know there's so much support but I wouldn't know where to start with it all. (Y02‐02)

Cultural norms around mental health influenced help‐seeking behaviour, with family attitudes persisting even when students recognised their own need for support. The same student remarked:

For my family, the whole mental health thing is not really a talking point. (Y02‐02)

In contrast to inconsistent formal support, peer networks proved vital. One student described a collaborative culture that countered individualising pressures in higher education:

We all really work together… we've built a really good support network. (Y04‐03)

The material reality of peer support was evident in accounts of friends providing support outside of the university to help with their studies:

He just starts banging on my door. Helps me get out of bed a lot of the times. (Y02‐02)

Peer mentoring provided crucial guidance, transmitting tacit knowledge that is rarely formally taught:

You'd have a port of call… two years above you… they'd give you little pointers. (Y04‐03)

However, not all students experienced this sense of belonging. One commuter student reflected on exclusion from the informal networks where crucial academic and social knowledge is shared:

I travel to and from… then go home. There's no sort of uni life for me. (Y03‐06)

## Discussion

This study revealed how biomedical science students at a post‐92 university with a strong widening participation mission navigated a landscape where engagement is shaped by pedagogical methods and support systems, not solely individual motivation. While students entered with intrinsic curiosity and altruistic aspirations, this motivation was contingent on subsequent educational experiences. For example, one student (Y02‐02) who expressed a deep ‘love’ for microbiology also described ‘dozing off’ during traditional lectures, while praising hands‐on simulations as ‘having fun while learning’. These contrasting accounts suggest that the pedagogical approach directly shaped whether intrinsic motivation was sustained or diminished. Progression was deeply influenced by the interplay of teaching practices, curriculum relevance and accessible support within structural constraints. For bioscience educators, understanding these dynamics is essential for designing inclusive learning environments that sustain engagement across a diverse student body.

A core finding was the direct pedagogical impact on motivation. Students' accounts aligned with expectancy‐value theory, showing that engagement was linked to the perceived value of the discipline [27]. Crucially, this value was activated or diminished by teaching methods. Hands‐on simulations were engaging and confidence‐building, while didactic lectures were widely described as disengaging. This finding aligns with experiential learning theory [28] and suggests the importance of active approaches in bioscience education [29]. For bioscience educators, integrating hands‐on laboratory simulations and enquiry‐based practicals is not merely enrichment but essential for sustaining motivation, particularly among students from diverse backgrounds. Notably, those who expressed the strongest intrinsic motivation were also most critical of passive teaching, suggesting that pedagogical disengagement may disproportionately affect the very students widening participation seeks to recruit. Motivation was further undermined by social isolation among commuting and mature students, highlighting how institutional culture and opportunities for integration sustain engagement, factors particularly important in bioscience disciplines where collaborative laboratory work is central.

The ‘support and belonging’ theme revealed a significant gap between institutional provision and student experience. While formal support existed, students struggled to navigate it, reflecting what has been termed the ‘hidden curriculum’ [30]. This refers to the unwritten, implicit norms and values transmitted through educational practices that advantage students with existing cultural capital while disadvantaging others. Students described knowing support was available but being unsure where to start, illustrating how widening participation students may lack tacit knowledge to mobilise resources, aligning with Bourdieu's concept of cultural capital [31]. In contrast, peer networks emerged as a vital source of academic and emotional support. Students described friends providing practical help with daily routines and peers normalising academic struggle by admitting shared confusion. These accounts demonstrate the importance of peer relationships for resilience and point to the need for support systems that formally integrate these organic communities [15, 32]. In bioscience education, this might involve structured near‐peer mentoring, collaborative laboratory groups and learning communities connecting students across year levels. The absence of competition noted by students, exemplified by the quote ‘There's never any competition. We're all happy for each other’ (Y04‐03), suggests peer networks can function as counterspaces [33] that protect students from seeing themselves as competitors in a system where grades and opportunities feel limited. This observation came directly from student accounts and is presented as a finding, not a recommendation. However, it does suggest that fostering collaborative learning environments could be invaluable for ensuring cohesiveness and student morale.

Curriculum design emerged as critical for innovation. Students found learning most fulfilling when it connected theory to professional practice, aligning with constructive alignment principles [34]. However, gaps in experiential learning constrained professional identity development, heightening employability anxiety [18]. Students described a Catch‐22 where lack of laboratory experience prevented them from securing the very opportunities needed to gain experience, showing how pandemic‐related disruptions have enduring consequences for students lacking alternative routes. For bioscience educators, this emphasises the importance of embedding authentic laboratory experiences throughout the curriculum and finding ways to develop practical skills when traditional laboratory access is limited. Effective approaches include flipped classroom models that replace passive lectures with active problem‐solving, simulation‐based learning using patient simulators or virtual laboratories, enquiry‐based practicals where students design and execute their own experiments and integrated case studies that explicitly link science content to clinical or professional scenarios. Such strategies have been shown to enhance engagement and reduce achievement gaps in biomedical education [35, 36, 37].

Students' expressed desire for greater involvement in shaping their learning aligns with the ‘students as partners’ model [38, 39], suggesting that curriculum codesign could be a productive approach to addressing the gaps they identified. In bioscience education, this could involve students codesigning laboratory protocols, contributing to practical assessment criteria or collaborating with staff to create resources for mastering scientific terminology. This aligns with Yosso's [33] community cultural wealth, positioning students' experiential knowledge as a resource for institutional improvement rather than a deficit to be remedied [33].

These educational factors are intensified by structural barriers. The themes revealed cumulative pressures where financial precarity, pandemic disruption and cultural exclusion compound one another [21, 40]. Accounts from mature students juggling childcare, illness and financial precarity while maintaining deadlines illustrate intersectional disadvantage [41]. Microaggressions recounted by participants revealed how gender, class and regional identity intersect to produce exclusionary experiences that students carry across institutions. For international students, mastering scientific language rather than everyday English reflects the hidden curriculum of disciplinary discourse [19]. Bioscience educators can address this by explicitly teaching disciplinary vocabulary, providing glossaries of key terms and designing activities that normalise learning scientific language. Consistent with widening participation research [42, 43], these findings confirm the persistence of financial and cultural barriers while emphasising how structural challenges are mediated by the quality of day‐to‐day educational practice.

Notably, participant experiences varied across year groups. First‐year students described transition challenges and scientific language acquisition, while second‐year students expressed growing anxiety about placement access. Final‐year and master's students both discussed heightened concerns about employability and career readiness. Such developmental differences between the different stages of years suggest that support interventions and pedagogical innovations may need to be tailored to specific stages of the student journey, rather than adopting a one‐size‐fits‐all approach.

Taken together, addressing inequities in biosciences education requires moving beyond institutional rhetoric to a holistic approach. It necessitates pedagogical innovation beyond the lecture hall and into the laboratory, support systems bridging formal and informal networks, and curricula codesigned for professional relevance. The transformative potential of such approaches is evident in students' accounts of foundation years as transformative and their appreciation for responsive staff, moments of institutional care starkly contrasting with experiences of marginalisation. While focused on biomedical science in a post‐92 context, the study demonstrates that fostering equitable progression depends on creating learning environments that actively recognise students' intersecting realities and value their contributions as partners. For bioscience educators, this means attending not only to who enters our programmes but to their daily experiences of pedagogy, curriculum and belonging once they arrive in lectures, laboratories and the informal spaces where scientific identity is forged.

## Limitations

This study has limitations. Self‐selection bias may have led to an overrepresentation of students with strong opinions, potentially excluding quieter voices. Focussing solely on a single post‐92 institution in a unique regional context limited the generalisability of findings to other UK universities. Uneven and sometimes small cohort sizes, along with the online format, may have constrained discussion and disproportionately weighted contributions from certain year groups. Additionally, perspectives from students who withdrew participation or left the programme were not captured, possibly omitting marginalised voices.

## Conclusion

This study suggests that equitable student progression requires moving beyond addressing structural barriers in isolation. The findings point to the importance of integrated learning systems where innovative, hands‐on pedagogies, codesigned curricula and responsive support systems work in concert. For bioscience educators, these findings suggest the importance of evidence‐led innovation across all dimensions of educational practice to foster genuine inclusion and widen participation. The voices of students themselves, those who described foundation years as ‘blessings’, who built peer networks to sustain each other, who aspired to work ‘at the highest level’, indicate that the capacity for success exists within widening participation cohorts. The question is whether institutions will create environments that recognise and nurture it.

## Conflict of interest

The authors declare conflict of interest.

## Author contributions

OJR, CR, PSF, BSL, DG, PD, KA‐A and TKS‐S acquired the data and performed formal analysis. OJR, CR, PSF, BSL, DG, PD, KA‐A, TKS‐S and AAK wrote and revised the manuscript. AAK conceived and designed the study, supervised the project and administered the research. All authors approved the final version for publication and agree to be accountable for all aspects of the work.

## Supporting information


**File S1.** COREQ (COnsolidated criteria for REporting Qualitative research) 32‐item checklist completed for this study.

**File S2.** Semi‐structured focus group topic guide, including motivation‐related prompts and discussion questions.

## Data Availability

The data that support the findings of this study are available from the corresponding author upon reasonable request. Due to the sensitive nature of the qualitative data, including potentially identifiable information from focus group participants, the data are not publicly available.
