# Supplementary material for: Pathways and pitfalls: a qualitative study of student experiences in biomedical science education
Source: FEBS Open Bio. 2026 Jun 8:10.1002/2211-5463.70285. Online ahead of print. doi: 10.1002/2211-5463.70285 (PMC13398928; doi:10.1002/2211-5463.70285)
Supplement: Supplementary file 2 — File S2. Semi‐structured focus group topic guide, including motivation‐related prompts and discussion questions. [file FEB4-9999-0-s001.pdf]

Focus Group Plan (45 Minutes)

Exploring Social and Systemic Barriers in Biomedical Science Education

1. **Welcome and Introductions (5 minutes)**

Facilitator's Introduction:

- Opening:
  "Hello everyone and thank you for taking the time to join this focus group. My name is [YOUR NAME] and I'll be leading today's session. The purpose of this discussion is to learn about your experiences as biomedical science students, particularly the challenges you may have faced on your journey toward becoming biomedical scientist graduates. Your insights will help identify barriers and inform strategies to better support students like you."
- Confidentiality Assurance:
  "I want to reassure you that everything you share today shall remain confidential and anonymous. Your input will be used only for research purposes and no identifying information will be included in any publications that may arise from discussions. Please feel free to share openly but only discuss what you're comfortable sharing."
- Session Structure:
  "The session will last 45-60 minutes. We'll begin with introductions, then move on to discussing challenges, support systems and solutions and finish by bringing things together.

Participant Introductions:

- "To start, I'd like to ask each of you to briefly introduce yourself and share why you chose to study biomedical science."

2. Setting Ground Rules (2 minutes)

- Facilitator to Participants:
  "Before we begin, we'll quickly go over a few ground rules to ensure a respectful and productive discussion:"
  1. "Respect each other's opinions and experiences."
  2. "Please give everyone the opportunity to speak."
  3. "Keep everything shared here confidential and within this group."

4. “Finally, remember that there are no right or wrong answers - all of your perspectives are valuable.”

- “Does anyone have any questions about these guidelines?”

3. Discussion Questions

Part 1: Exploring Challenges (15 minutes)

- Facilitator Introduction:
  “We’ll start by discussing the challenges you may have faced on your journey to becoming a biomedical scientist.”
- Question 1:
  “What challenges have you faced outside of your studies in your journey toward becoming a biomedical scientist?”
  (Probe if needed: Are there any financial, social, or systemic barriers you’ve encountered?)
- Question 2:
  “Do you feel that factors like socio-economic background or access to opportunities have influenced your experience as a student? If so, how?”
- Question 3:
  “Are there any specific challenges you think might be unique to students from underrepresented groups in biomedical science?”

Part 2: Support and Resources (10 minutes)

- Facilitator Introduction:
  “Next, let’s talk about the support systems or resources that have been helpful or could be improved.”
- Question 4:
  “What kinds of support (e.g., mentorship, peer networks, or external resources) have been most helpful to you in overcoming challenges?”
- Question 5:
  “Are there any forms of support or opportunities you wish were more accessible to students like you?”
- Question 6:
  “Can you share any strategies or resources you’ve found particularly useful in navigating barriers?”

Part 3: Aspirations and Solutions (10 minutes)

- Facilitator Introduction:
  "Finally, let's discuss your motivations and ideas for improving diversity and support in biomedical science."
- Question 7:
  "What motivates you to continue working toward becoming a biomedical scientist?"
- Question 8:
  "What changes or initiatives do you think could help students from diverse or underrepresented backgrounds succeed in biomedical science?"
- Question 9:
  "How do you think increasing diversity and representation in biomedical science could benefit the field and healthcare as a whole?"

4. Conclusion (3 minutes)

- Final Question:
  "Before we finish, is there anything else you'd like to share about your experiences or ideas for supporting students in becoming biomedical scientists?"
- Closing Remarks:
  - "Thank you all for sharing your experiences and insights today. Your feedback is incredibly valuable and will help inform strategies to support biomedical science students and improve diversity in the field."
  - "As a reminder, everything shared here will remain anonymous, but if you have additional thoughts after today's session, feel free to contact me"
